# Voxel-Based Morphometry in Individuals at Genetic High Risk for Schizophrenia and Patients with Schizophrenia during Their First Episode of Psychosis

**DOI:** 10.1371/journal.pone.0163749

**Published:** 2016-10-10

**Authors:** Miao Chang, Fay Y. Womer, Chuan Bai, Qian Zhou, Shengnan Wei, Xiaowei Jiang, Haiyang Geng, Yifang Zhou, Yanqing Tang, Fei Wang

**Affiliations:** 1 Department of Radiology, The First Affiliated Hospital of China Medical University, Shenyang, Liaoning, PR China; 2 Brain Function Research Section, The First Affiliated Hospital of China Medical University, Shenyang, Liaoning, PR China; 3 Department of Psychiatry, The First Affiliated Hospital of China Medical University, Shenyang, Liaoning, PR China; 4 Department of Psychiatry, Washington University School of Medicine, St. Louis, Missouri, United States of America; 5 Department of Psychiatry, Yale University School of Medicine, New Haven, Connecticut, United States of America; Nathan S Kline Institute, UNITED STATES

## Abstract

**Background:**

Understanding morphologic changes in vulnerable and early disease state of schizophrenia (SZ) may provide further insight into the development of psychosis.

**Method:**

Whole brain voxel-based morphometry was performed to identify gray matter (GM) regional differences in 60 individuals with SZ during their first psychotic episode (FE-SZ), 31 individuals at genetic high risk for SZ (GHR-SZ) individuals, and 71 healthy controls.

**Results:**

Significant differences were found in several regions including the prefrontal cortex, parietal lobe, temporal lobe, hippocampus, occipital lobe, and cerebellum among the three groups (p<0.05, corrected). Compared to the HC group, the FE-SZ group had significantly decreased GM volumes in several regions including the cerebellum, hippocampus, fusiform gyrus, lingual gyrus, supramarginal gyrus, and superior, middle, and inferior temporal gyri and significantly increased GM volumes in the middle frontal gyrus and inferior operculum frontal gyrus (p<0.05). The GHR-SZ group had significant decreases in GM volumes in the supramaginal gyrus, precentral gyrus, and rolandic operculum and significant increases in GM volumes in the cerebellum, fusiform gyrus, middle frontal gyrus, inferior operculum frontal gyrus, and superior, middle, and inferior temporal gyri when compared to the HC group (p<0.05). Compared to the GHR-SZ group, the FE-SZ group had significant decreases in GM volumes in several regions including the cerebellum, fusiform gyrus, supramarginal gyrus, and superior, middle, and inferior temporal gyri (p<0.05).

**Conclusions:**

The findings herein implicate the involvement of multisensory integration in SZ development and pathophysiology. Additionally, the patterns of observed differences suggest possible indicators of disease, vulnerability, and resiliency in SZ.

## Introduction

Neural abnormalities develop and progress long before the onset of the clinical hallmarks of schizophrenia (SZ) [1]. This challenges efforts to implement effective treatment that substantially improve the clinical outcomes of affected individuals, pushing earlier identification and intervention in SZ to the forefront in psychiatric research. There are many questions as to the markers of SZ development, the appropriate timing and targets of such interventions, and potential resiliency factors. Understanding the neural underpinnings of SZ particularly during the vulnerability period and initial stages of the illness is critical in the search for answers to these questions.

Structural and functional abnormalities in multiple brain regions, including the prefrontal cortex, anterior cingulate cortex, hippocampus, thalamus, and cerebellum, have been observed in both SZ and high risk populations, implicating disruptions in various neural networks in the development and pathophysiology of SZ [1–4]. While studies have increasingly focused on neural alterations in individuals at high risk for (HR-SZ), studies comparing high-risk individuals and those during early stages of SZ are relatively limited. Such studies would provide direct comparison between high-risk and disease populations with less confounding effects from psychotropic medications and illness chronicity. Moreover, they could implicate candidate markers of vulnerability and progression to SZ, as well as potential markers for resiliency to the disorder. Prior studies in HR-SZ suggest that abnormalities in the prefrontal cortex (PFC) and temporal cortex are involved in conversion to psychosis and that other regions such as the parietal cortex, anterior cingulate cortex, and hippocampus may also confer vulnerability to SZ [3, 5]. However, interpretation of findings from these studies is complicated by the varying criteria used to define HR-SZ, including those based on clinical symptoms or family history. Differences in altered regions have been observed when comparing individuals at clinical high-risk for SZ (CHR-SZ) and those at genetic high risk (GHR-SZ) [5, 6]. Studies have reported up to 30–35% conversion to psychosis during multi-year follow-up in CHR-SZ, however these results may be partly attributed to sampling strategies and reflect the magnitude of subsyndromal symptoms that are already present within these populations [7]. Furthermore, there is still significant debate regarding which clinical symptoms or features would be more specific and predictive of psychosis conversion in CHR-SZ. Criteria for individuals at genetic high risk for SZ (GHR-SZ) can be more clearly defined. SZ is highly heritable with estimated heritability rate of 81% [8]. Recent genome-wide association studies suggest that some specific genes such as NRGN, CACNA1C and ZNF804A have been identified risk loci for SZ, which associated with brain structure and function, and illness development in SZ [9–11]. Nevertheless, genetic factors appear to have substantial influence on brain structure and function, and illness development in SZ. A recent study by Nenadic et al. compared two high risk groups- those with GHR and those with high but sub-clinical psychotic symptom profiles. Their findings suggest that GHR-SZ is more similar to individuals during their first episode of SZ (FE-SZ) in brain morphologic changes than CHR-SZ to FE-SZ [6]. Subsequently, studies of GHR-SZ may more reliably yield potential neural markers of SZ development and resiliency.

In this study, we examined structural brain alterations in GHR-SZ and FE-SZ using whole brain voxel-based morphometry (VBM). GHR-SZ was defined as offspring of at least one parent with schizophrenia.

## Methods

### Participants

Participants in this study included 60 FE-SZ and 31 GHR-SZ individuals, and 71 healthy controls (HC). FE-SZ and GHR-SZ participants were recruited from the outpatient clinics of the Department of Psychiatry, First Affiliated Hospital of China Medical University, Shenyang, China, and HC participants were recruited from Shengyang, China by advertisement. The absence or presence of Axis I disorders were independently assessed by 2 trained psychiatrists using the Structured Clinical Interview for DSM-IV Axis I Disorders (SCID) for participants over 18 years old and the Schedule for Affective Disorders and Schizophrenia for School-Age Children-present and Lifetime Version (K-SADS-PL) for participants under 18 years old. FE-SZ participants met Diagnostic and Statistical Manual of Mental Disorders, Fourth Edition (DSM-IV) diagnostic criteria for schizophrenia, schizophreniform disorder, or schizoaffective disorder, and no other Axis I disorders. GHR-SZ participants were offspring of individuals with schizophrenia (at least one parent with SZ and were not related to the FE-SZ participants) who were within the peak period for illness onset (late adolescence to 30 years old) but did not meet criteria for psychotic disorder or any other DSM-IV Axis diagnoses. HC participants did not have current or lifetime Axis I disorder or history of psychotic, mood, or other Axis I disorders in first-degree relatives (as determined detailed family history). For all participant groups, individuals were excluded for history of substance or alcohol abuse or dependence, head injury, neurologic or concomitant major medical disorder, and any MRI contraindications for MRI. This study was approved by the Institutional Review Board of China Medical University, and all participants provided written informed consent after detailed description of the study.

Symptom measures using the Brief Psychiatric Rating Scale (BPRS) and medication history were obtained in the FE-SZ and GHR-SZ groups. In the FE-SZ group, 27 were taking atypical antipsychotics including clozapine, risperidone, olanzapine, aripiprazole and quetiapine, 1 was taking perphenazine, and 32 were not taking any psychotropic medication at the time of scan. Only 1 GHR-SZ participant had prior exposure to sertraline for 1 month for social phobia symptoms but was not taking any psychotropic medications at the time of scan. All antipsychotic doses were converted to chlorpromazine equivalents using standard procedures [12] (Table 1).

**Table 1:** Participant demographic and clinical characteristics.

	HC (n = 71)	GHR-SZ (n = 31)	FE-SZ (n = 60)	Statistic value	*p* value (2 Tailed)
**Age (years)**	20.63 ± 3.48 (14–25)	18.39 ± 3.37 (13–24)	18.32 ± 3.09 (13–25)	F = 9.52	<0.001
**Gender (M / F)**	27 / 44	21 / 10	29 / 31	χ^2^ = 27.66	<0.05
**Medication at the time of scan (Y / N)**	N/A	1* / 30	28 / 32	χ^2^ = 17.76	<0.001
**Medication-CPZ Equivalent**	-	-	223.86±193.51	N/A	N/A
**Duration of illness (months)**	N/A	N/A	8.06 ± 14.02	N/A	N/A
**BPRS total score**	N/A	15.52 ± 6.51	36.58 ± 12.78	t = 51.75	<0.001

HC: healthy controls; GHR-SZ: genetic high-risk schizophrenia; FE-SZ: first-episode schizophrenia; BPRS: Brief Psychiatric Rating Scale.

Age is expressed as means ± standard deviations (SD) with range in parentheses. Duration of illness and BPRS score are expressed as means ± SD.

### Image Acquisition

MRI was performed on a GE Signa HDX 3.0T magnetic resonance (MR) scanner using a T1-weighted, 3D fast spoiled gradient-echo (FSPGR) sequence with the following parameters: TR/TE = 7.2/3.2ms, Flip = angle 13°, field of view (FOV) = 240×240mm^2^, 176 slices, voxel size = 1mm^3^.

### Data processing

Processing was performed using the DARTEL algorithm Statistical Parametric Mapping software (SPM8, http://www.fil.ion.ucl.ac.uk/spm/software/spm8/) under the MATLAB R2010b platform (Mathworks, Sherborn, MA, USA). Briefly, the segmentation function was used to divide the regions into gray matter (GM), white matter (WM), and cerebrospinal fluid (CSF) using the ‘new segment’ tool implemented in SPM8. During spatial normalization, inter-subject registration was achieved using respective registration based on group assignment. A modulation step was used to ensure that the overall amount of tissue in a class was unaltered. The segmented images were normalized to the Montreal Neurological Institute (MNI) template and were smoothed with an 8-mm full width at half-maximum (FWHM) Gaussian filter. The voxel size of data acquisition was 1mm^3^ and the voxel size of normalized data was 1.5mm^3^.

### Statistical analysis

Statistical analyses for demographic and clinical characteristics were performed using IBM SPSS Statistics for Windows, Version 21.0 (Armonk, NY, USA). (Table 1)

Whole brain GM volumetric comparisons among the diagnostic groups were performed using a full-factorial design, with age and gender as covariates, using SPM8 (Wellcome Trust Centre for Neuroimaging, http://www.fil.ion.ucl.ac.uk/spm/software/spm8/). Statistical significance was determined by a voxel-level statistical threshold (p<0.01) with AlphaSim correction for multiple comparisons (minimum cluster size of 444 voxels). AlphaSim correction was performed using DPABI software (DPABI_V1.2_141101, http://rfmri.org/dpabi). Post hoc comparisons between groups were performed for significant GM regions. Values were extracted from all significant regions in the three group analysis and analyzed in pair-wise two sample t-tests (HC vs. SZ, HC vs. GHR-SZ, GHR-SZ vs. SZ) using SPSS. Statistical significance was determined by p<0.05. Analyses were also performed to test for effects of demographic and clinical variables on GM volumes for significant regions from the three-group comparison (S1 File).

## Results

### Demographics and clinical characteristics

Demographic and clinical characteristics for all groups are presented in Table 1. Age significantly differed among the diagnostic groups [Degrees of freedom (df) = 2, F = 9.52, p<0.001] with post-hoc comparisons showing that the GHR-SZ and FE-SZ groups were significantly younger than the HC group. There were also significant differences in gender among the groups (df = 2, χ^2^ = 7.66, p<0.05). Subsequently, all analyses were controlled for age and gender.

BPRS scores were significantly different between the GHR-SZ and FE-SZ groups (df = 1, t = 51.75, p<0.001) with higher mean score in the FE-SZ group, as expected. Medication exposure was also significantly different between these two groups (df = 1, χ^2^ = 17.76, p<0.001) with a higher percentage of FE-SZ participants (53%) taking psychotropic medications compared to the GHR-SZ group (3%). Results from subsequent analyses, as reported below, remained unchanged with inclusion of BPRS scores and medication exposure.

### Whole brain GM VBM analyses

#### Three group comparison

**Fig 1:**
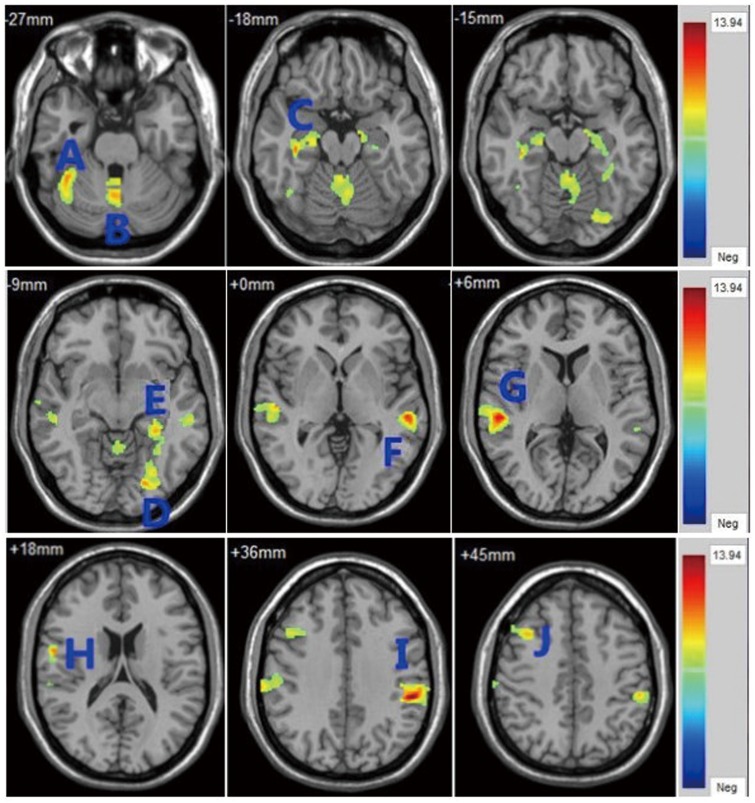
Clusters showing significant differences in the three group analysis (GHR-SZ, FE-SZ, and HC). GHR-SZ: genetic high-risk schizophrenia; FE-SZ: first-episode schizophrenia; HC: healthy controls. Letters represent clusters of significant difference in the three group analysis and correspond to regions listed in Table 2.

Significant differences were found in several regions including the prefrontal cortex, parietal lobe, temporal lobe, hippocampus, occipital lobe, and cerebellum among the three groups (df = 2, p<0.05, corrected). (Table 2 and Fig 1)

**Table 2:** Clusters and corresponding regions showing significant differences in the three group analysis (GHR-SZ, FE-SZ, and HC).

Cortical Regions	BA	Cluster Size	MNI Coordinates			F Values
			X	Y	Z	
A R Cerebellum Anterior Lobe	37	589	40	-49	-25	7
R Cerebellum Posterior Lobe		505				
R Fusiform Gyrus		198				
R Inferior Temporal Gyrus		20				
B L Cerebellum Posterior Lobe		456	3	-61	-28	6.77
Vermis		149				
C R Hippocampus	28/36/20/3	267	37	-24	-18	6.56
R ParaHippocampal Gyrus		147				
R Fusiform Gyrus		43				
D L Fusiform Gyrus	18/19	317	-21	-79	-10	7.81
L Lingual Gyrus		188				
L Inferior Occipital Gyrus		113				
E L Hippocampus	37/19/28/36/34/35	291	-30	-39	-7	7.63
L ParaHippocampal Gyrus		212				
L Fusiform Gyrus		151				
L Lingual Gyrus		15				
F L Middle Temporal Gyrus	22/21	658	-57	-28	-1	5.63
L superior Temporal Gyrus		134				
G R superior Temporal Gyrus	22/42/41/21/40/13/43	1535	55	-28	7	6.51
R Middle Temporal Gyrus	2/6/4/3/40/43/1/44	266				
H R Supra Maginal Gyrus	40/2/3/1/42/4	392	55	-3	18	8.86
R Precentral Gyrus		230				
I L Superior Parietal Lobule		747	-51	-34	34	9.38
L Supra Marginal Gyrus	9月8日	717				
L Postcentral Gyrus		80				
L Superior Temporal Gyrus		21				
J R Middle Frontal Gyrus		525	34	16	43	9.2

GHR-SZ: genetic high-risk schizophrenia; FE-SZ: first-episode schizophrenia; HC: healthy controls. Letters represent clusters of significant difference in the three group analysis, which are shown in Fig 1. BA: Brodmann Area. MNI: Montreal Neurological Institute.

#### FE-SZ and HC

**Fig 2:**
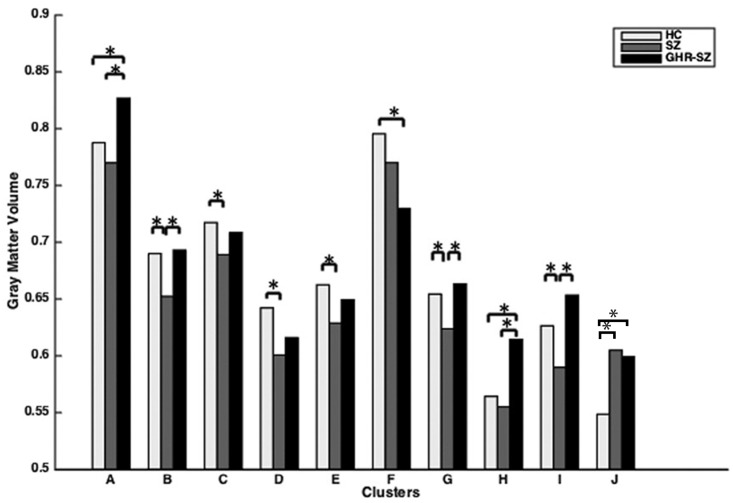
Post-hoc, pair-wise comparison of clusters showing significant differences in the three group analysis (GHR-SZ, FE-SZ, and HC). GHR-SZ: genetic high-risk schizophrenia; FE-SZ: first-episode schizophrenia; HC: healthy controls. Letters represent clusters of significant difference in the three group analysis and correspond to regions listed in Table 2. * indicates p<0.05.

The FE-SZ group had significantly decreased GM volumes in the left cerebellum posterior lobe, vermis, bilateral hippocampi, bilateral parahippocampal gyri, bilateral fusiform gyri, left lingual gyrus, left inferior occipital gyrus, right middle temporal gyrus, bilateral superior temporal gyri, right rolandic operculum, left inferior parietal lobule, left postcentral gyrus, and left supramarginal gyrus and significantly increased GM volumes in the right middle frontal gyrus and inferior operculum frontal gyrus, compared to the HC group (df = 1,p<0.05). (Fig 2)

#### GHR-SZ and HC

When compared to the HC group, the GHR-SZ group demonstrated significant decreases in GM volumes in the right supramaginal gyrus, right precentral gyrus, and right rolandic operculum and significant increases in GM volumes in the right cerebellum anterior lobe, right cerebellum posterior lobe, right fusiform gyrus, right inferior temporal gyrus, left middle temporal gyrus, superior temporal gyrus, right middle frontal gyrus, and right inferior operculum frontal gyrus (df = 1, p<0.05). (Fig 2)

#### FE-SZ and GHR-SZ

Significant decreases in GM volumes were observed in bilateral cerebellum posterior lobe, right cerebellum anterior lobe, vermis, right fusiform gyrus, right inferior temporal gyrus, right middle temporal gyrus, right superior temporal gyrus, right rolandic operculum, bilateral supramarginal gyri, bilateral precentral gyri, left inferior parietal lobule, and left superior temporal gyrus in the FE-SZ group compared to the GHR-SZ group (df = 1, p<0.05). (Fig 2)

There were no significant effects of demographic and clinical variables on GM volumes (S1 and S2 Tables).

## Discussion

Consistent with the converging view of SZ as a disorder with widespread neural disruptions, multiple regions including the prefrontal, temporal, and parietal cortices, hippocampus, and cerebellum were found to have significant differences when comparing GHR-SZ, FE-SZ, and HC individuals in this study [1, 5, 13]. Post-hoc comparisons of these regions with significant differences suggest potential indicators of disease, vulnerability, and resiliency or compensatory mechanisms in SZ, although these interpretations are tentative and warrant further investigation. The FE-SZ group demonstrated significantly decreased GM volumes in the cerebellar vermis, right superior temporal gyrus, right middle temporal gyrus, right operculum rolandic, left inferior parietal lobule, and left postcentral gyrus when compared to the GHR-SZ and HC groups, suggesting that these are altered regions in the disease state. The FE-SZ group also showed significantly decreased GM volumes in the bilateral hippocampus, bilateral parahippocampal gyri, bilateral fusiform gyri, left lingual gyrus, and left inferior occipital gyrus when compared to the HC group but not to the GHR-SZ group. These regions were not significantly different between the GHR-SZ and HC groups, and GM volumes for these regions in the GHR-SZ group appeared intermediate to the FE-SZ and HC groups. This may reflect changes related to disease vulnerability that progress with disease onset. Significant increases in GM volumes were observed in the right middle frontal gyrus and right inferior operculum frontal gyrus in the FE-SZ and GHR-SZ group, compared to the HC group. These may be markers of vulnerability to SZ and possibly reflect neuroinflammation, which may be present during vulnerability and early disease states in SZ [4, 14]. Significantly increased GM volumes were also found in the right cerebellum anterior and posterior lobes, right fusiform gyrus, right inferior temporal gyrus, right suparmarginal gyrus, right precentral gyrus, and right operculum rolandic in the GHR-SZ group when compared to the FE-SZ and HC groups. These increases could reflect resiliency or compensatory mechanisms against SZ development, as well as neuroinflammation with disease vulnerability. The implications for the significantly decreased GM volumes in the left superior and middle temporal gyri in the GHR-SZ group compared to the HC group are less clear. GM volumes of these regions in FE-SZ were intermediate to the HC and GHR-SZ group though no significant differences were observed compared to either the HC or GHR-SZ groups. It is possible that we were underpowered to detect significant differences between the HC and FE-SZ groups in these regions. Additionally, there may be multiple or more complex processes underlying these findings, such as the interaction between neuroprotection and neural dysfunction. The mechanism underlying this finding remains incompletely understood and warrants further investigation.

The findings in this study are consistent with those from prior studies of HR-SZ and FE-SZ, including direct comparisons of these groups, particularly with observations of altered frontotemporal and parietal regions in these samples; however there are some discrepancies involving regions such as the cingulate cortex, thalamus, and basal ganglia and in hemispheric lateralization that may be attributed to differences in ethnic background, sample size, definition of HR-SZ, medication exposure, and methodological techniques [3, 5, 6, 13, 15–20]. In general, our findings indicate abnormalities in frontal, temporal, and parietal regions involved in language, sensory processing, and somatosensory integration in SZ, as well as alterations in the cerebellum, for which there is mounting evidence of its role in cognitive processes in SZ in addition to its known roles in motor coordination and sensory integration [21–24]. This is of particular interest as prior studies suggest impaired multisensory integration (MSI) involving these regions in the development and pathophysiology of SZ[25–28]. Individuals with SZ appear to have selective deficits in audiovisual integration of linguistic stimuli, and the severity of MSI deficits may correspond with the modalities of hallucinations in SZ [25, 27, 29]. Postural sway, which reflects cerebellar function and integration of proprioceptive, vestibular, and visuals inputs, has been shown to be increased in SZ and HR-SZ, and appears to correlate with negative symptoms in HR-SZ[26, 30, 31]. These findings regarding MSI in SZ, while limited, are not surprising given the convergent evidence for the central role of structural and functional dysconnectivity in the disorder[13, 32, 33].

There are limitations to the present study. The study was cross-sectional and could not examine changes related to psychosis conversion in GHR-SZ. Such observations would better elucidate neural markers that predict psychotic onset and differentiate from alterations reflecting vulnerability but not necessarily progression to disease onset in this population. Clinical characterization was limited in this study, and we were unable to correlate our findings with specific clinical variables. BPRS scores did not appear to have significant effects on the findings. Future investigations should include more extensive clinical and behavioral data including cognitive testing. Potential confounding effects by medication exposure and illness duration were minimized in the study; however 47% of the FE-SZ participants received antipsychotics prior to scanning. The mean duration of illness was 8 months in the FE-SZ group. Additional analyses did not reveal significant effects of medication and illness duration within each patient group on regional brain volume (S2 Table). However, we did not collect patient data regarding duration of medication exposure, which may be an important characteristic for future studies. Moreover, age and gender are key variables showing between-group differences across the clinical groups. Age and gender could therefore be related to brain maturity and confound observed across-group clinical differences. We therefore performed analysis to to test for interactions between age and group and gender and group for effects in regions identified in our three-group comparison analyses. We did find that there were significant interaction effects in the vermis and bilateral middle/superior temporal gyri for the group×age interaction and vermis for group×gender interaction, which previous studies have also reported [34–36] (S1 Table). Although most regions did not show significant interaction effects, future experiments that explicitly control for demographic within the same cross-sectional designs are needed to validate the present findings.

In summary, significant differences were found in multiple brain regions including the frontal, temporal, and parietal cortices as well as the hippocampus, occipital cortex, and cerebellum in comparison of GHR-SZ, FE-SZ, and HC groups in this study. Potential markers of vulnerability, disease, and resiliency or compensatory mechanisms could be inferred based on the pattern of observed differences, however further studies are needed for more definitive conclusions. Additionally, our findings in GHR-SZ and FE-SZ support implications from other studies of impaired MSI in SZ vulnerability and development. If MSI indeed is importantly involved in progression to SZ onset, studies of MSI in SZ may lead to novel and promising strategies for prevention and early intervention in high risk and affected individuals such as cognitive remediation or enhancement therapy targeting sensory integration[37].

## Supporting Information

S1 Fig Clusters showing significant differences in the three-group analysis (GHR-SZ, FE-SZ, and HC). p<0.001, cluster size = 218 (p<0.05 corrected). GHR-SZ: genetic high-risk schizophrenia; FE-SZ: first-episode schizophrenia; HC: healthy controls. Number labels represent clusters of significant difference in the three-group analysis and correspond to regions listed in S1 Table.
(TIF)

S1 File Supporting Information. Part 1: Relationship between gray matter volume and clinical information. Part 2: Detailed on narrowing threshold of whole brain GM VBM analyses.
(DOCX)

S1 Table The effects of age and gender on GM in healthy control, genetic high risk for schizophrenia and first episode schizophrenia groups. T value (P value). Results considered statistically significant at p<0.05 corrected. *p < 0.05 false discovery rate corrected. GM: Gray matter.
(DOCX)

S2 Table Correlations between clinical characteristics and GM volume in healthy control, genetic high risk for schizophrenia and first episode schizophrenia groups. T value(P value). *p< 0.05 false discovery rate corrected. GM = Gray matter; HC: healthy control; GHR-SZ: genetic high risk for schizophrenia; FE-SZ: first episode schizophrenia BPRS = Brief Psychiatric Rating Scale.
(DOCX)

S3 Table Clusters and corresponding regions showing significant differences between GHR-SZ, FE-SZ, and HC groups. GHR-SZ: genetic high-risk schizophrenia; FE-SZ: first-episode schizophrenia; HC: healthy controls. BA: Brodmann Area. MNI: Montreal Neurological Institute.
(DOCX)
