# Correction: Voxel-Based Morphometry in Individuals at Genetic High Risk for Schizophrenia and Patients with Schizophrenia during Their First Episode of Psychosis

**DOI:** 10.1371/journal.pone.0170146

**Published:** 2017-01-09

**Authors:** Miao Chang, Fay Y. Womer, Chuan Bai, Qian Zhou, Shengnan Wei, Xiaowei Jiang, Haiyang Geng, Yifang Zhou, Yanqing Tang, Fei Wang

Table 2 is incorrect. The column titled “BA” has numerous errors. The 8^th^, 7^th^, and 4^th^ line from the bottom should be the 7^th^, 5^th^, and 1^st^ line from the bottom respectively. Additionally, the 4^th^ line from the bottom incorrectly reads “9月8日.” It should read “9/8” in the 1^st^ line from the bottom. The correct version of Table 2 can be seen here.

**Table 2:** Clusters and corresponding regions showing significant differences in the three group analysis (GHR-SZ, FE-SZ, and HC). GHR-SZ: genetic high-risk schizophrenia; FE-SZ: first-episode schizophrenia; HC: healthy controls. Letters represent clusters of significant difference in the three group analysis, which are shown in Figure 1. BA: Brodmann Area. MNI: Montreal Neurological Institute.

Cortical Regions	BA	Cluster Size	MNI Coordinates			*F* Values
			X	Y	Z	
**A** R Cerebellum Anterior Lobe	37	589	40	-49	-25	7
R Cerebellum Posterior Lobe		505				
R Fusiform Gyrus		198				
R Inferior Temporal Gyrus		20				
**B** L Cerebellum Posterior Lobe		456	3	-61	-28	6.77
Vermis		149				
**C** R Hippocampus	28/36/20/3	267	37	-24	-18	6.56
R ParaHippocampal Gyrus		147				
R Fusiform Gyrus		43				
**D** L Fusiform Gyrus	18/19	317	-21	-79	-10	7.81
L Lingual Gyrus		188				
L Inferior Occipital Gyrus		113				
**E** L Hippocampus	37/19/28/36/34/35	291	-30	-39	-7	7.63
L ParaHippocampal Gyrus		212				
L Fusiform Gyrus		151				
L Lingual Gyrus		15				
**F** L Middle Temporal Gyrus	22/21	658	-57	-28	-1	5.63
L superior Temporal Gyrus		134				
**G** R superior Temporal Gyrus	22/42/41/21/40/13/43	1535	55	-28	7	6.51
R Middle Temporal Gyrus		266				
**H** R Supra Maginal Gyrus	2/6/4/3/40/43/1/44	392	55	-3	18	8.86
R Precentral Gyrus		230				
**I** L Superior Parietal Lobule	40/2/3/1/42/4	747	-51	-34	34	9.38
L Supra Marginal Gyrus		717				
L Postcentral Gyrus		80				
L Superior Temporal Gyrus		21				
**J** R Middle Frontal Gyrus	9/8	525	34	16	43	9.2
