# Supplementary figures and images for: Voxel-Based Morphometry in Individuals at Genetic High Risk for Schizophrenia and Patients with Schizophrenia during Their First Episode of Psychosis
Source: PLoS One. 2016 Oct 10;11(10):e0163749. doi: 10.1371/journal.pone.0163749 (PMC5056757; doi:10.1371/journal.pone.0163749)

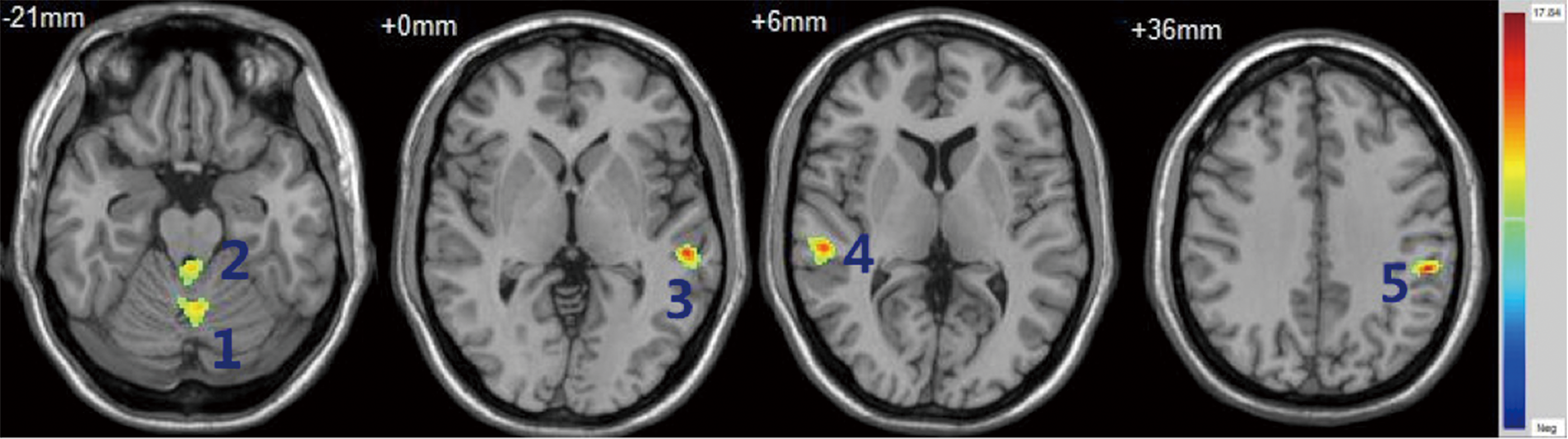

Supplement: S1 Fig — p<0.001, cluster size = 218 (p<0.05 corrected). GHR-SZ: genetic high-risk schizophrenia; FE-SZ: first-episode schizophrenia; HC: healthy controls. Number labels represent clusters of significant difference in the three-group analysis and correspond to regions listed in S1 Table. (TIF) [file pone.0163749.s001.tif]
