# Supplementary material for: Voxel-Based Morphometry in Individuals at Genetic High Risk for Schizophrenia and Patients with Schizophrenia during Their First Episode of Psychosis
Source: PLoS One. 2016 Oct 10;11(10):e0163749. doi: 10.1371/journal.pone.0163749 (PMC5056757; doi:10.1371/journal.pone.0163749)
Supplement: S1 File — Part 1: Relationship between gray matter volume and clinical information. Part 2: Detailed on narrowing threshold of whole brain GM VBM analyses. (DOCX) [file pone.0163749.s002.docx]

**Supporting Information**

**Part 1: Relationship between gray matter volume and clinical information**

**Methods**

Univariate analyses of variance were performed to test for potential group by age and group by gender interactions in regions with significant volume differences identified in the three group comparison [healthy controls (HC), genetic high risk for schizophrenia (GHR-SZ), first episode schizophrenia (FE-SZ)]. Partial correlation analyses (two-tailed) were performed in the GHR-SZ and FE-SZ, controlling for age and gender, to explore the relationship between the gray matter (GM) volumes and Brief Psychiatric Rating Scale (BPRS) total scores. Additional analyses were performed to test for medication effects for overall medication status (medicated vs. un-medicated). Additional partial correlation analyses were also performed to test for associations between GM volume and medication dosage, as well as illness duration in FE-SZ. Results were considered statistically significant at p<0.05 false discovery rate corrected.

**Results**

There was a significant age by group and gender by group interaction effect in the vermis. There was also a significant age by group effect in the middle/superior temporal gyrus. No other regions showed significant interaction effects between diagnosis and age or diagnosis and gender in (S1Table). There were no significant correlations between BPRS total scores and GM volumes in either GHR-SZ or FE-SZ group (S2 Table). There were no significant effects of medication and illness duration in GM volume in FE-SZ (S2 Table).

**Part 2: Detailed on narrowing threshold of whole brain GM VBM analyses**

**Methods**

Statistical significance of whole brain grey matter (GM) volumetric comparisons among the diagnostic groups was determined by a voxel-level statistical threshold (p<0.001) with AlphaSim correction (p<0.05) for multiple comparisons (minimum cluster size of 218 voxels)(DPABI_V1.2_141101, http://rfmri.org/dpabi).

**Results**

Significant differences were found in several regions including the parietal lobe, temporal lobe and cerebellum among the three groups (Degrees of freedom =2, p<0.001, cluster size=218) (S3 Table and S1Figure).
