# Supplementary material for: Voxel-Based Morphometry in Individuals at Genetic High Risk for Schizophrenia and Patients with Schizophrenia during Their First Episode of Psychosis
Source: PLoS One. 2016 Oct 10;11(10):e0163749. doi: 10.1371/journal.pone.0163749 (PMC5056757; doi:10.1371/journal.pone.0163749)
Supplement: S1 Table — T value (P value). Results considered statistically significant at p<0.05 corrected. *p < 0.05 false discovery rate corrected. GM: Gray matter. (DOCX) [file pone.0163749.s003.docx]

**S1 Table**

| Regions | Age by diagnosis interactions | Gender by diagnosis interactions |
| --- | --- | --- |
|  |  |  |
| A | 1.383(0.250) | 1.426(0.237) |
| B | 5.564(0.001)* | 6.506(0.000)* |
| C | 0.981(0.403) | 0.529(0.663) |
| D | 2.069(0.107) | 0.618(0.604) |
| E | 3.165(0.026) | 3.833(0.011) |
| F | 9.488(0.000)* | 1.172(0.322) |
| G | 8.460(0.000)* | 2.233(0.087) |
| H | 0.922(0.432) | 1.846(0.141) |
| I | 3.272(0.023) | 0.625(0.600) |
| J | 0.552(0.648) | 0.762(0.517) |
