# Supplementary material for: Voxel-Based Morphometry in Individuals at Genetic High Risk for Schizophrenia and Patients with Schizophrenia during Their First Episode of Psychosis
Source: PLoS One. 2016 Oct 10;11(10):e0163749. doi: 10.1371/journal.pone.0163749 (PMC5056757; doi:10.1371/journal.pone.0163749)
Supplement: S2 Table — T value(P value). *p< 0.05 false discovery rate corrected. GM = Gray matter; HC: healthy control; GHR-SZ: genetic high risk for schizophrenia; FE-SZ: first episode schizophrenia BPRS = Brief Psychiatric Rating Scale. (DOCX) [file pone.0163749.s004.docx]

| **Brain Regions** | **Comparison of medicated vs. un-medicated participants** | **Correlations between medication dosage and regional GM volumes** | **Correlations between illness duration and regional GM volumes** | **Correlations between BPRS and regional GM volumes** | | |
| --- | --- | --- | --- | --- | --- | --- |
|  | **FE-SZ** | **FE-SZ** | **FE-SZ** | **HC** | **GHR-SZ** | **FE-SZ** |
| A | -0.449(0.655) | 0.083(0.688) | -0.084(0.533) | 0.062(0.779) | 0.228 (0.243) | -0.149（0.273） |
| B | 0.234(0.816) | 0.046(0.825) | -0.064(0.633) | 0.390(0.066) | 0.198(0.313) | -0.104(0.445) |
| C | 0.748(0.458) | -0.088(0.668) | -0.060(0.654) | -0.115(0.602) | 0.251(0.197) | 0.108(0.429) |
| D | -0.138(0.891) | -0.304(0.131) | 0.081(0.545) | 0.502(0.015) | -0.048(0.807) | 0.077(0.571) |
| E | -0.316(0.753) | -0.188(0.357) | 0.005(0.968) | 0.023(0.915) | 0.387(0.042) | 0.130(0.340) |
| F | 0.217(0.829) | -0.220(0.280) | -0.158(0.237) | 0.249(0.251) | 0.165(0.402) | -0.102(0.454) |
| G | 0.678(0.500) | -0.043(0.833) | 0.009(0.949) | 0.224(0.305) | 0.188(0.337) | 0.120(0.379) |
| H | 0.535(0.595) | -0.187(0.360) | -0.129(0.333) | 0.380(0.074) | 0.233(0.233) | 0.086(0.527) |
| I | -0.347(0.730) | -0.473(0.015) | -0.096(0.473) | -0.105(0.633) | 0.290(0.134) | 0.098(0.470) |
| J | -0.287(0.775) | 0.314(0.118) | -0.072(0.592) | 0.158(0.471) | 0.086(0.664) | 0.067(0.625) |

**S2 Table**
