# Supplementary material for: Voxel-Based Morphometry in Individuals at Genetic High Risk for Schizophrenia and Patients with Schizophrenia during Their First Episode of Psychosis
Source: PLoS One. 2016 Oct 10;11(10):e0163749. doi: 10.1371/journal.pone.0163749 (PMC5056757; doi:10.1371/journal.pone.0163749)
Supplement: S3 Table — GHR-SZ: genetic high-risk schizophrenia; FE-SZ: first-episode schizophrenia; HC: healthy controls. BA: Brodmann Area. MNI: Montreal Neurological Institute. (DOCX) [file pone.0163749.s005.docx]

| Brain Regions | BA | Cluster Size | MNI Coordinates | | | F Values |
| --- | --- | --- | --- | --- | --- | --- |
|  |  |  | X | Y | Z |  |
| 1 Bilateral Cerebellum Anterior Lobe |  | 506 | 1.5 | -61.5 | -27 | 10.44 |
| Bilateral Cerebellum Posterior Lobe |  |  |  |  |  |  |
| Vermis |  |  |  |  |  |  |
| 2 Bilateral Cerebellum Anterior Lobe |  | 232 | 0 | -40.5 | -22.5 | 9.57 |
| Vermis |  |  |  |  |  |  |
| 3 L Middle Temporal Gyrus | 21/22 | 424 | -57 | -28.5 | -1.5 | 11.61 |
| 4 R Superior Temporal Gyrus | 41/42/22 | 349 | 57 | -28.5 | 7.5 | 11.57 |
| R Middle Temporal Gyrus |  |  |  |  |  |  |
| 5 L Inferior Parietal Lobule | 40/2/1 | 243 | -51 | -34.5 | 34.5 | 11.78 |
| L Supra Marginal Gyrus |  |  |  |  |  |  |

**S3 Table**
